# Mapping cerebellar subregional volumes and heterogeneity in schizophrenia spectrum disorders and violence

**DOI:** 10.1007/s00406-025-02081-1

**Published:** 2025-09-24

**Authors:** Thomas Fischer-Vieler, Jaroslav Rokicki, Milin Kim, Esten Leonardsen, Thomas Wolfers, Christina Bell, Gabriela Hjell, Natalia Tesli, Nina Bang, Ingrid Melle, Ole A. Andreassen, Christine Friestad, Petter Andreas Ringen, Unn K. Haukvik

**Affiliations:** 1 NORMENT Norwegian Centre for Mental Disorders Research and Institute of Clinical Medicine, University of Oslo, Oslo, Norway; 2 SIFER Centre of Research and Education in Forensic Psychiatry, Oslo University Hospital, Oslo, Norway; 3 Division of Mental Health and Addiction, Oslo University Hospital, Oslo, Norway; 4 Department of Psychology, Faculty of Social Sciences, University of Oslo, Oslo, Norway; 5 Department of Psychiatry and Psychotherapy, Tübingen, Center for Mental Health, University of Tübingen and German Center for Mental Health (DZPG), Tübingen, Germany; 6 Østfold Hospital Trust, Graalum, Norway; 7 Norwegian University of Science and Technology (NTNU), Trondheim, Norway; 8 Vestre Viken Hospital Trust, Drammen, Norway

**Keywords:** Psychotic disorders, Schizophrenia, Violence, Structural imaging, Cerebellum, Normative modelling

## Abstract

Cerebellar abnormalities have been linked to schizophrenia and aggressive behaviour. Subregional cerebellar morphology reflects structural organization and function and show great heterogeneity, which may be linked to shared or distinct underlying mechanisms of violence and psychosis but remain unexplored in these marginalized groups. Total and subregional cerebellar volumes were estimated from 3 T MRI scans from persons with schizophrenia spectrum disorders without (SSD-NV; n = 107) or with (SSD-V; n = 36) a history of severe violence, violent offenders without schizophrenia (NPV; n = 20), and 411 healthy controls. Group difference analyses by GLMs were complemented by novel heterogeneity analyses using pre-trained models and norm-charts of lifespan cerebellar volumes to assess individual deviation patterns. We found decreased grey matter volumes in the posterior cerebellar hemispheres and the vermal regions in both SSD-V and SSD-NV, but with a different subregional distribution. Specifically, SSD-V showed differences in the right Crus I (*p* = .045, Cohen’s d = .62) and Vermis IX (*p* < .001, Cohen’s d = .81) in the groupwise comparisons. In addition, individual-level extreme negative deviations were detected in the Vermis IX region in 11% of SSD-V and in some subregions of the posterior cerebellum and the vermis in > 10% of NPV. By applying novel analytical tools, we report different patterns of cerebellar subregion volume reductions associated with violence in individuals with or without schizophrenia spectrum disorders. The partly converging results from the group comparisons and the normative modelling analyses demonstrate the usefulness of complemental methodological approaches to disentangle the complex biological associations between violence and psychosis.

## Introduction

Despite that the majority of persons with schizophrenia spectrum disorders (SSDs) never engage in violence, several studies indicate that SSDs are associated with an increased risk of violent behavior [1, 2]. This association remains even when controlling for historical and clinical risk factors for violence, e.g., substance abuse [1], and poses both a considerable clinical and societal challenge [3, 4].

Several neuroimaging studies have investigated the brain structural and functional correlates of violence in psychosis, where the most consistent findings include decreased volumes of the hippocampus, amygdala, and regions within the prefrontal cortex (see [5, 6] for review). However, studies show mixed results which may reflect the influence of methodological choices, cohort differences, and region of interest selection. A brain structure that deserves a closer focus in this context, as it seems to play an important role in both psychosis [7] and violence [8], is the cerebellum. Though the cerebellum is reported to contain as much as 80% of the neurons in the human brain [9, 10], little interest had been devoted to exploring its functions beyond modulating motoric processes until Schmahmann postulated the “Cerebellar Cognitive Affective Syndrome” [11] in 1998. At the same time, Andreasen introduced the concept of cognitive dysmetria [12] and ascribed the cerebellum a pivotal role when proposing a unitary model of schizophrenia [13]. Since then, several structural and functional MRI studies have explored the cerebellum’s involvement in a wide range of cognitive, social, and emotional processes [14–17]. Already the early imaging studies using CT scans showed relatively consistent pathology of the cerebellar vermis in persons with schizophrenia [18]. Furthermore, a decrease in cerebellar grey matter was shown in the very early stages of the illness and before any medication was established [19]. Cerebellar grey matter volume decrease, especially in the posterior hemisphere, is one of the most consistently reported cerebral structural changes in schizophrenia [7, 20].

Furthermore, owing to findings linking the cerebellum to the modulation of aggression and impulsivity in healthy individuals [21–23], there has been a growing interest in exploring cerebellar structure in conditions characterized by psychopathic traits and a low threshold to violent behaviour [24]. In violent offender populations, grey matter volume decrease was most consistently present in the right posterior cerebellar hemisphere, the vermis, and the whole cerebellum [8]. Moreover, cerebellar volume decreases in the more inferior subregions have been strongly associated with rule-breaking behaviour in individuals with conduct disorder and similar conditions [25].

However, studies exploring cerebellar volume in schizophrenia spectrum patients with a history of violence compared to non-violent patients are scarce. To our knowledge, the only two structural MRI studies directly comparing these groups have yielded inconclusive results. One did not report any volume differences of the whole cerebellum between men with schizophrenia with or without a history of violence [26], while the other, performing voxel-based morphometry, found decreased grey matter bilaterally in men with schizophrenia and a history of violence [27]. As both SSDs and violence constitute highly complex phenomena, groupwise comparisons of cerebellar volume may conceal widespread individual-level volumetric heterogeneity [28, 29]. Indeed, novel methods in neuroimaging, such as normative modelling, provide a framework for investigating brain deviations from the norm [30]. Importantly, this approach moves beyond regular case–control group comparisons to capture abnormalities and volumetric heterogeneity at the individual level. The basic principle of normative modelling are paediatric growth charts where selected individual body measures are plotted against their normal distribution which enables the identification of patterns of extreme variations. A recently published paper by our group describes the potential of normative modelling in forensic psychiatry neuroimaging research using study populations mostly overlapping with the current study [31]. One of the main findings where extreme (Z > 2) volume decrease of the right cerebellar cortex in 10.5% of the violent schizophrenia spectrum disorder patients.

The aim of the present study was to explore and compare the structure of cerebellar subregions, i.e. the lobules of the cerebellum, across a sample of thoroughly assessed patients with schizophrenia spectrum disorders with (SSD-V) and without (SSD-NV) a history of severe violence and healthy controls (HC). Furthermore, we aimed to investigate if there were volume alterations with a specific signature in a group of non-psychotic violent offenders (NPV) compared to the three groups mentioned above.

Finally, we sought to map out the extent of individual differences within groups by performing normative modelling analyses [30, 32]. This approach takes into consideration the biological heterogeneity of clinical constructs such as schizophrenia spectrum diagnoses and trait violence and may add valuable information to case–control designs [33].

We hypothesized global cerebellar volume reductions in the two patient groups and the violent offenders, which would be most extensive in the SSD-V group and least pronounced in the NPV group (i). Furthermore, we expected the main volume differences between the groups to be in the posterior lobules, which are reported to have an important function in modulating higher cognitive processes [20] (ii). Finally, we hypothesized that analyses of cerebellar subregional volumes at the individual level (not group level as in hypothesis i and ii) would partly be overlapping with the group level analyses, but also capture more subtle volume alterations than conventional group level analyses (iii).

## Materials and methods

### Sample

SSD patients with a history of severe violence (SSD-V; n = 36), i.e., murder, attempted murder, severe physical assault, inclusive sexual assaults, were included from high-security psychiatric wards at Oslo University Hospital and Østfold Hospital Trust, Norway, between 2018 and 2021. SSD patients without a history of violence (SSD-NV; n = 107) were recruited from major psychiatric hospitals and their outpatient clinics in Oslo, Norway. The subject sample is partly overlapping our previous MRI studies exploring hippocampus [34], amygdala [35] and hypothalamic nuclei volumes [36].

Inclusion criteria for both groups were schizophrenia spectrum disorders (schizophrenia, schizophreniform disorder, schizoaffective disorder) according to the Diagnostic and Statistical Manual of Mental Disorders Fourth Edition [37], age between 18 and 65 years, Norwegian language knowledge to understand the study protocol and procedures, IQ scores above 65, and the ability to give informed consent to study participation.

The violent offenders without comorbid schizophrenia (NPV; n = 20) consisted of incarcerated persons serving a preventive detention sentence in a high security prison in the Oslo region, Norway, due to perpetration of an act of severe violence.

Additional inclusion criteria for the SSD-V and NPV participants were safety evaluations regarding study procedures and permission to leave the hospital ward/prison for the MRI acquisition. Exclusion criteria for all groups were head trauma leading to loss of consciousness for more than 10 min and somatic illness that might have affected brain morphology.

The non-violent, non-psychotic healthy control (HC) group (n = 411) consisted of persons with no history of severe mental disorder and was randomly selected from the Norwegian national population registry (https://www.ssb.no/en). All were residents of the Oslo region, Norway, and they were invited by a personal letter to participate in the study. Only men were included in all groups since few females serve preventive detention in Norway, and no female participants could be recruited to the NPV group and only a few to the SSD-V group.

Written informed consent was obtained from all participants. The work was conducted in accordance with the Declaration of Helsinki. The study is approved by the Regional Ethics Committee, the Norwegian Directory of Health and the Norwegian Data Protection Authority.

### Clinical assessments

All assessments were performed by trained psychologists or medical doctors. Diagnoses were assessed using the Structured Clinical Interview for DSM-IV axis 1 disorders (SCID-I) [37].

The assessment of violence was based on a thorough examination of court files, criminal and hospital records. The inclusion of SSD-V and SSD-NV patients was decided by consensus of at least one experienced forensic psychiatrist (TF-V, PAR, CB) and an experienced researcher in this field (UKH). Patients were omitted if there was any doubt about the severity of a violent act.

Psychotic symptoms were assessed using the Positive and Negative Syndrome Scale (PANSS) [38]. Depressive symptoms were assessed by the PANSS depressive symptoms subscore [39]. Alcohol use was investigated by the AUDIT (Alcohol Use Disorders Identification Test) [40], and illegal drug use by the DUDIT (Drug Use Disorders Identification Test) [41]. Insight was assessed by the Birchwood Insight Scale (BIS) [42]. Use of antipsychotic medication was calculated based on Defined Daily Dosages (DDD) in accordance with guidelines from World Health Organization (https://www.whocc.no/atc_ddd_index/).

Psychopathy traits were assessed with the Hare Psychopathy Checklist-Revised (PCL-R) [43] based on interviews, court documents, and/or medical records.

### MRI-acquisition and post processing

MRI data were acquired using two GE 3 T scanners. The MRI data obtained before the upgrade were collected on a 3 T GE Signa HDxt scanner (GE Medical Systems, Milwaukee, WI, USA) using a standard 8-channel head coil at Oslo University Hospital, Norway. T1-weighted volumes were acquired using a sagittal 3D fast spoiled gradient echo (FSPGR) sequence with the following parameters: repetition time (TR) 7.8 ms, echo time (TE) 2.9 ms, flip angle 12°, slice thickness 1.2 mm, 166 slices, field of view (FOV) 256 mm × 256 mm, acquisition matrix 256 × 192 mm, reconstructed in-plane resolution 256 × 256 mm. MRI data after the upgrade were collected on a 3 T GE 750 Discovery scanner using a 32-channel head coil at Oslo University Hospital. T1-weighted volumes were acquired using a sagittal 3D BRAVO sequence with the following parameters: repetition time (TR) 8.2 ms, echo time (TE) 3.2 ms, flip angle 12°, slice thickness 1.0 mm, 192 slices, field of view (FOV) 256 mm × 256 mm. All MRI scans were evaluated by a neuroradiologist to ensure no brain pathology affecting the analyses.

T1-weighted MRI volumes were pre-processed using the standard FreeSurfer recon-all pipeline (version 7.1; http://surfer.nmr.mgh.harvard.edu/). Image quality control progressed in two steps. First, all T1w images were processed with MRIQC [44]. The images classified by a default machine learning algorithm to an exclude node with a probability of at least 0.5 were further visually investigated by two trained raters (NT and JR). In the second step, quality assessment of the area and thickness of cortical maps was performed by a careful visual inspection of lateral and medial snapshots of all maps by the same raters. The participant was excluded if the surface values included negative values, uncharacteristic patterns or strong value disbalance between hemispheres.

For segmentation, we used the ACAPULCO version 0.2.1 algorithm [45], which is a state-of-the-art cerebellum parcellation algorithm. The algorithm divides the cerebellum into 28 lobules, including bilateral Lobules I–VI; Crus I and II; Lobules VIIB, VIIIA, VIIIB, and IX-X; Vermis VI, VII, VIII, IX, and X; and Corpus Medullare (CM). It also calculates volume (mm^3^) of each region.

We used pretrained normative models of the cerebellum [32]. Briefly, models were trained using data of 27,117 individuals aged 3–85 from 132 sites (age mean = 54.36, SD = 20.31, 47% male) for voxel wise and lobular cerebellar data. These models were trained using a Bayesian linear regression framework with likelihood warping using the sinarcsinsh function [46]. We modelled scanner and age effects on the cerebellar volume. The volumetric information was preprocessed using ACAPULCO [47] and condensed into the 28 cerebellar lobules. Models were both trained and applied to our data, these models are shared in the https://github.com/amarquand/PCNtoolkit. Further details on normative models are described in [32]. An additional harmonization step was implemented to account for the absence of scanner data used to acquire data in the model. An unmatched group of healthy controls (n = 1003) was utilized to fine-tune model parameters, enabling the application of normative models to unseen sites.

### Statistical analyses

Statistical analyses were conducted using IBM SPSS Statistics for Windows, Version 29.0.0.0 (IBM Corp., 2022).

Continuous variables were tested for normality by analyzing normality plots. Group comparisons for continuous normally distributed variables were evaluated with independent sample *t*-tests, group comparisons for continuous data with skewed distributions were evaluated with Mann–Whitney U-tests, and group comparisons for dichotomous data were evaluated with chi-squared tests (Table 1).

**Table 1:** Sample characteristics

	SSD-V (N = 36)		SSD-NV (N = 107)		NPV (N = 20)		HC (N = 411)		Test	*p*-value
	Mean (SD)	Range	Mean (SD)	Range	Mean (SD)	Range	Mean (SD)	Range		
Age at MR	35.14 (8.79)	19.16–54.15	29.52 (8.64)	18.73–57.76	42.44 (14.43)	22.75–70.95	35.25 (8.69)	18.04–56.40	F = 17.27	*p* < 0.001
IQ	94.00 (15.51)	67–118	101.80 (13.76)	69–133	101.59 (12.77)	82–128	114.91 (9.82)	76–138	F = 56.70	*p* < 0.001
PANSS positive	16.75 (7.58)		13.99 (5.05)						T = 2.39	*p* = .018
PANSS negative	17.70 (7.24)		16.54 (6.15)							NS
PANSS general	30.75 (9.68)		32.07 (8.76)							NS
PANSS depression	9.81 (4.36)		11.91 (4.22)							NS
BIS	6.73 (2.69)		7.90 (2.36)						T = 2.27	*p* = .025
AUDIT	4.81 (4.59)	0–16	6.58 (6.38)	0–27			5.77 (3.27)	0–22		NS
DUDIT	9.47 (10.08)	0–34	5.67 (8.96)	0–37			.42 (1.66)	0–17	χ^2^ = 119.7	*p* < .001
DDD	1.66 (0.74)		1.23 (0.84)						T = 2.54	*P* = .013
PCL-R	18.51	8.85			20.84	7.84				NS

SSD-V: schizophrenia spectrum disorder with a history of severe violence; SSD-NV: non-violent schizophrenia spectrum disorder; NPV: non-psychotic violent offenders; HC: healthy controls; SD: standard deviation; NS: non-significant; PANSS: Positive and Negative Syndrome Scale; BIS: Birchwood Insight Scale, sum score; AUDIT: Alcohol Disorders Identification Test; DUDIT: Drug Disorders Identification Test; DDD: defined daily dose; PCL-R: Psychopathy Check List Revised, total score

For the group analyses, we controlled for extreme univariate outliers (Z-value > 4 on any lobule volume) and removed five individuals (three HC and two SSD-NV). As an introductory exploration we conducted ANCOVA with the total cerebellar volume as the dependent variable, the four participant groups as the independent, and age, estimated intracranial volume (ICV) and scanning site as covariates (Table 2). We added the volume of the cerebellar lobules up to seven superior anatomical sections [48] to hold the number of comparisons within reasonable limits. To assess group differences, first, we performed MANCOVA with the seven sections as the dependent variables and age, estimated intracranial volume (ICV) and scanning site as covariates (Table 3). We adjusted for the number of the seven sections using a conservative Bonferroni correction.

**Table 2:** Total cerebellum: Pairwise comparisons, corrected for FDR (q = .05). 6 unique comparisons

Region	Group 1	Mean volume mm^3^	SD	Group 2	Mean volume mm^3^	SD	*p* adjusted	Cohen’s d
Tot cerebellum	HC	153,597	10,989	SSD-NV	147,887	12,399	< .001	.49
Tot cerebellum	HC	153,597	10,989	SSD-V	145,529	14,150	.006	.64
Tot cerebellum	HC	153,597	10,989	NPV	146,500	14,572	.426	
Tot cerebellum	NPV	146,500	14,572	SSD-NV	147,887	12,399	.567	
Tot cerebellum	NPV	146,500	14,572	SSD-V	145,529	14,150	.469	
Tot cerebellum	SSD-V	145,529	14,150	SSD-NV	147,887	12,399	.919

**Table 3:** MANCOVA regions, Bonferroni corrected

Region	*p*	Partial Eta^2^
Left anterior	.336	.014
Right anterior	.49	.012
Left superior posterior	< .007	.034
Right superior posterior	< .007	.041
Left inferior posterior	.266	.015
Right inferior posterior	.091	.019
Vermis	< .007	.030

Box’s test .201. Wilks’ Lambda .007. Pillai’s Trace .007. Levene’s test not significant

Anterior Lobe: Lobules I-III, IV, V

Superior Posterior Lobe: Lobules VI, VIIB, Crus I, Crus II

Inferior Posterior Lobe: Lobules VIIIA, VIIIB, IX, X

Vermis: Vermis VI, VII, VIII, IX, X

Three individuals from the SSD-NV group were excluded from the analysis because of Mahalanobis distance beyond the critical value (dM > 24.32). The single lobules within the sections that showed significant differences between the groups (Table 4) were then explored with a second MANCOVA, again with ICV, age and scanning site as covariates (Table 5). Three individuals were flagged as multiple outliers (one HC and two PSY-NV, dM > 34.53) and excluded from further analyses. Homogeneity of covariance in the MANCOVAs was estimated with Box’s test while homogeneity of variances between the groups in the ANCOVAs was tested with Levene’s test.

**Table 4:** Pairwise comparisons regions, corrected for FDR (q = .05). 42 unique comparisons. 10 pairs with lowest *p* adjusted

Region	Group 1	Mean volume mm^3^	SD	Group 2	Mean volume mm^3^	SD	*p* adjusted	Cohen’s d
Left Sup Post	HC	44,374	3741	SSD-NV	42,646	3827	< .001	.46
Right Sup Post	HC	43,994	3701	SSD-NV	42,557	3905	< .001	.38
Vermis	HC	6860	628	SSD-V	6430	736	.017	.62
Vermis	HC	6860	628	SSD-NV	6599	664	.032	.40
Right Sup Post	HC	43,994	3701	SSD-V	41,588	4249	.050	.61
Left Sup Post	HC	44,374	3741	SSD-V	42,078	4885	.070	
Left Anterior	HC	7516	794	SSD-NV	7292	864	.078	
Right Anterior	HC	8724	907	SSD-NV	8503	925	.084	
Left Inf Post	HC	14,054	1634	SSD-NV	13,533	1778	.121	
Right Inf Post	HC	13,199	1447	NPV	12,256	1696	.122	

HC: healthy controls; SSD-NV: non-violent Schizophrenia spectrum disorder; SSD-V: Schizophrenia spectrum disorder with a history of severe violence; NPV: Non-psychotic violent offenders; SD: standard deviation; Anterior: Anterior Lobe: Lobules I-III, IV, V;

Sup Post: Superior Posterior Lobe: Lobules VI, VIIB, Crus I, Crus II; Inf Post: Inferior Posterior Lobe: Lobules VIIIA, VIIIB, IX, X;

Vermis: Vermis VI, VII, VIII, IX, X

**Table 5:** Pairwise comparisons lobules, corrected for FDR (q = .05). 78 unique comparisons. 15 pairs with lowest *p* adjusted

Region	Group 1	Mean volume mm^3^	SD	Group 2	Mean volume mm^3^	SD	*p*	Cohen’s d
Left Crus I	HC	16,374	2002	SSD-NV	15,745	1989	.039	.32
Left Crus I	HC	16,374	202	SSD-V	15,243	1983	.092	
Left Crus II	HC	9933	1486	SSD-NV	9507	1470	.078	
Left VI	HC	11,019	1334	SSD-NV	10,592	1516	.091	
Right VI	SSD-NV	10,061	1516	NPV	10,558	1821	.117	
Right Crus I	HC	16,732	1927	SSD-V	15,579	1772	.045	.62
Right Crus I	HC	16,732	1927	SSD-NV	16,398	1974	.084	
Right Crus II	HC	10,355	1550	SSD-NV	9651	1482	< .001	.46
Vermis VIII	HC	2361	314	SSD-NV	2234	318	.049	.40
Vermis VIII	HC	2361	314	SSD-V	2206	367	.200	
Vermis IX	HC	1258	165	SSD-NV	1186	178	< .001	.42
Vermis IX	HC	1258	165	SSD-V	1117	181	< .001	.81
Vermis X	HC	370	59	SSD-NV	348	55	.031	.39
Vermis X	HC	370	59	SSD-V	339	39	.086	
Vermis X	NPV	353	75	SSD-V	339	39	.208	

Box’s test .163. Pillai’s Trace < .001 Wilks’ Lambda < .001. Leven’s test significant for right VIIB and left VIIB

HC: healthy controls;SSD-NV: Non-violent schizophrenia spectrum disorder; SSD-V: Schizophrenia spectrum disorder with a history of severe violence;NPV: Non-psychotic violent offenders; SD: standard deviation

To account for multiple comparisons, a False Discovery Rate (FDR) correction was performed [49].

To control for potential effects of alcohol, medication, psychopathic traits, depression and insight, we re-ran the analyses with AUDIT as a measure for alcohol consumption, defined daily dosages (DDD) for antipsychotic medication, PCL-R total score for psychopathic traits, PANSS depression score for depression symptoms and BIS score for insight added as covariates in separate models.

To summarize inter-individual variation within each group (SSD-V, SSD-NV, NPV and HC), we computed deviation scores (Z-scores) by separating them into positive and negative deviations. The percentage of subjects within each group with extreme positive (Z > 2) and negative (Z < -2) deviations at a given region were compared and reported [50].

## Results

### Clinical and demographical characteristics

Clinical and demographic statistics are summarized in Table 1. The groups differed in age, illicit substance use (DUDIT), and IQ. The SSD-V group had the lowest IQ score and the highest DUDIT score, while the SSD-NV was the youngest group. Furthermore, there was significantly higher score for positive symptoms, higher doses of antipsychotic medication, and less insight in the SSD-V group compared to SSD-NV group. There were no other significant group differences.

### Group differences in cerebellar and subregional volumes

Total cerebellar volume was significantly lower in both SSD-NV (*p* < 0.001, Cohen’s d = 0.49) and SSD-V (*p* = 0.006, Cohen’s d 0.64) compared to HC (Table 2).

In the SSD-V patients, there were significant volume decreases in right Crus I (*p* = 0.045, Cohen’s d = 0.62), and Vermis IX (*p* < 0.001, Cohen’s d = 0.81) compared to HC.

**Fig. 1:**
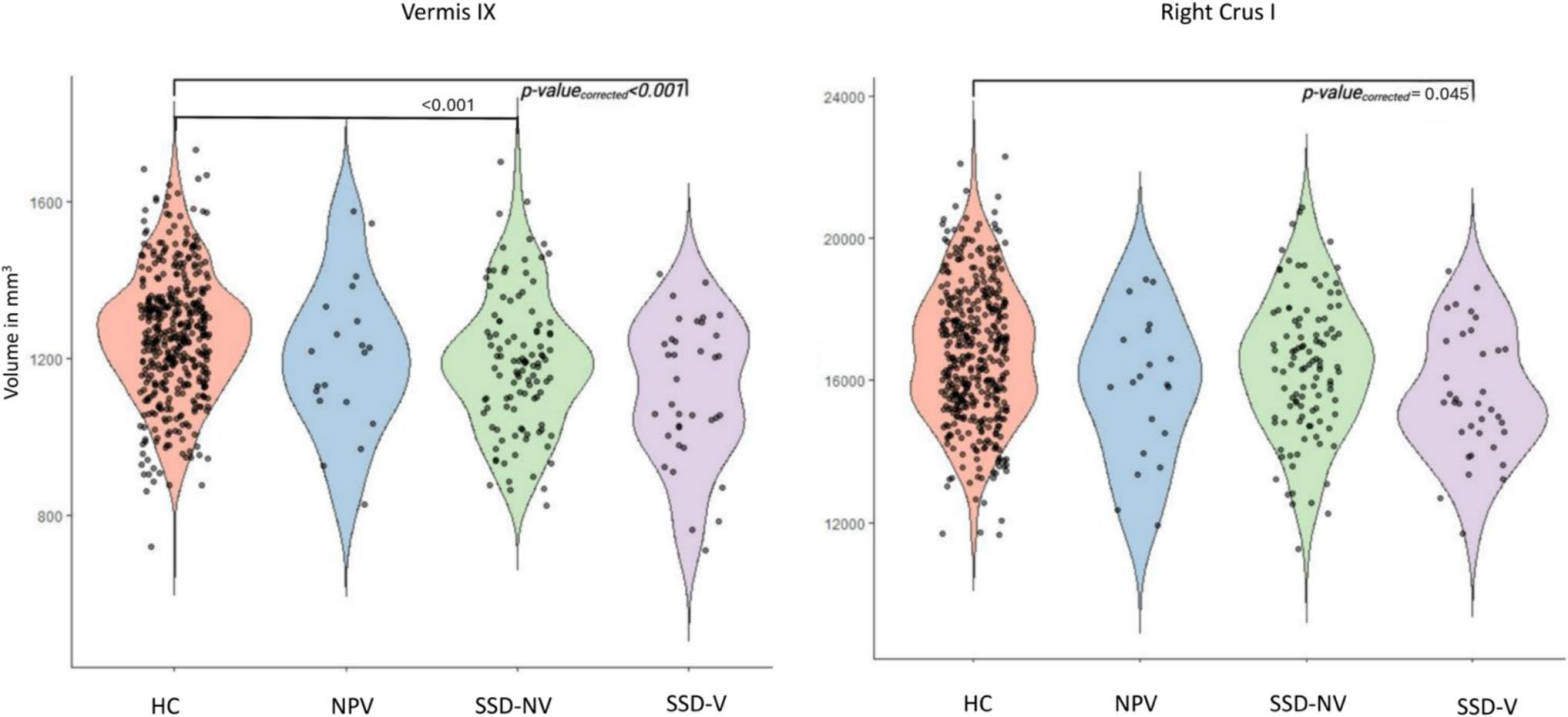
Violine plots showing volume distributions of the subregions Vermis IX and right Crus I. One dot represents one individual

**Fig. 2:**
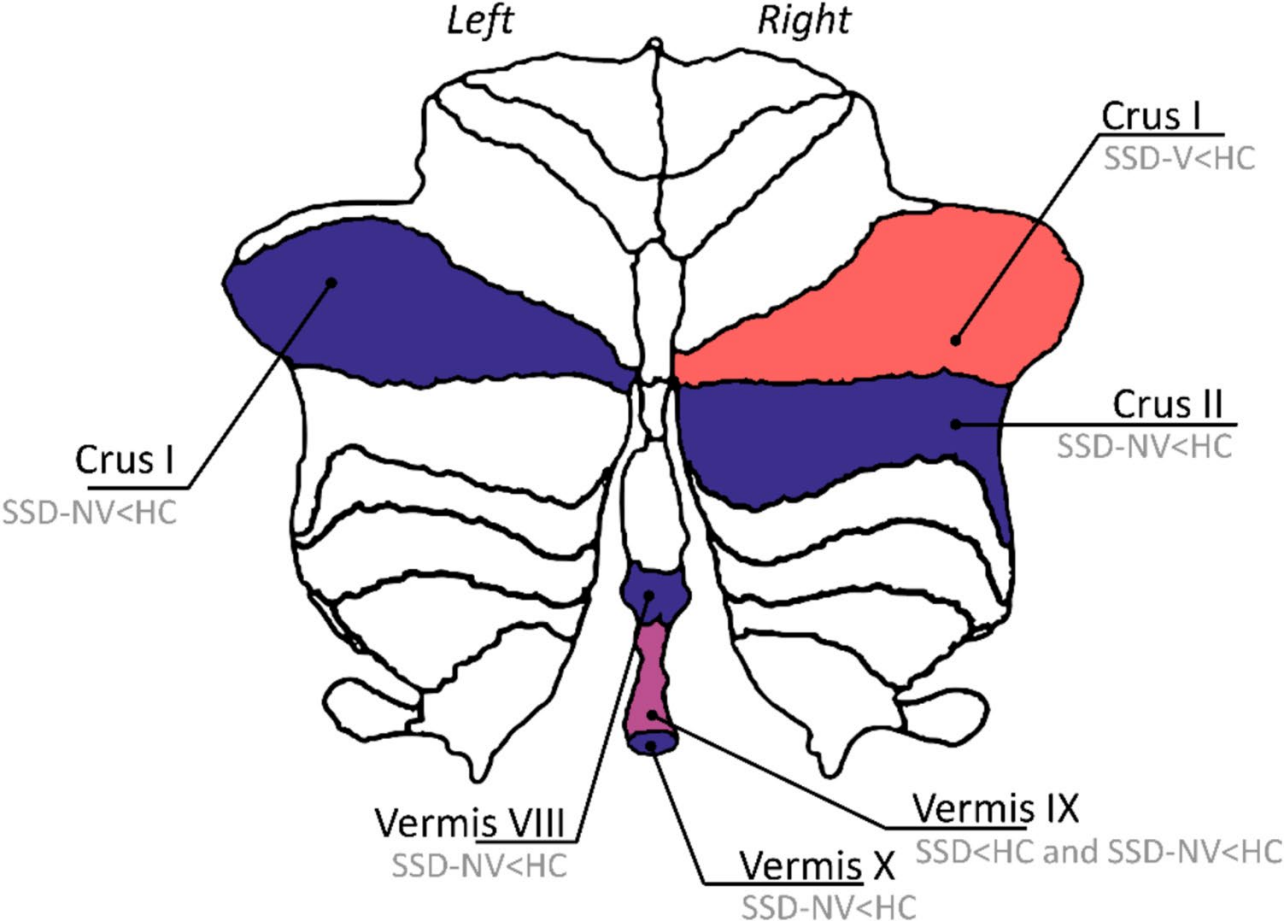
Flatmap of the cerebellum depicting patterns of significant volume decreases in the SSD-V group (red), and the SSD-NV group (blue), respectively, compared to healthy controls

SSD-NV patients had significantly lower volumes in the following cerebellar lobules in comparison to HC: left Crus I (*p* = 0.039, Cohen’s d = 0.32), right Crus II (*p* < 0.001, Cohen’s d = 0.46), Vermis VIII (*p* = 0.049, Cohen’s d = 0.40), Vermis IX (*p* < 0.001, Cohen’s d = 0.42), and Vermis X (*p* = 0.031, Cohen’s d = 0.39) (Tables 3, 4, 5, Figs. 1 and 2).

There were no significant differences between SSD-NV and SSD-V, or between NPV and the other groups.

Alcohol consumption (AUDIT: Pillai’s trace = 0.12, F = 2.28, *p* < 0.001, no substantial impact on p-values in the pairwise comparisons), Antipsychotic medication (DDD: Pillai’s trace = 0.07, F = 0.59, *p* = 0.86), psychopathic traits (PCL-R total: Pillai’s trace = 0.54, F = 1.46, *p* = 0.23), depression symptoms (PANSS depression Pillai’s trace 0.17, F = 0.67, *p* = 0.77), and insight (BIS: Pillai’s trace = 0.11, F = 1.01, *p* = 0.45) did not show significant impact on the volumes of the cerebellar lobules.

### Individual-level cerebellar and subregional volumes

The normative modelling analyses revealed more than 10% extreme negative outliers in the Vermis IX region of the SSD-V group (11.1%). In the NPV group, extreme negative deviations in more than 10% of the individuals were found in left lobule VIII B (15%), right lobule X (20%), Vermis VI (15%), Vermis X (15%), and the whole cerebellum (15%) (Table 5). Seven NPV individuals had at least one extreme negative deviation, while only one had deviations in all the lobules mentioned above.

Extreme positive deviations were found in 15% of the NPV individuals bilaterally in lobule VI (Table 6). No extreme deviations were exceeding 10% of the individuals in the SSD-NV group and the HC, respectively (Table 6 and 7).

**Table 6:** Positive deviations (> 2 Z) in percent

Lobule	HC		SSD-NV	SSD-V	NPV
Left Crus I	5.07		2.75	0	5.00
Left Crus II	3.38		2.75	5.56	0
Left I-III	0.24		0	2.78	0
Left IV	3.62		2.75	0	5.00
Left IX	1.93		0.92	0	0
Left V	0.48		1.83	0	0
Left VI	0.24		0.92	2.78	**15.00**
Left VIIB	2.17		1.83	0	5.00
Left VIIIA	1.69		0.92	2.78	0
Left VIIIB	2.42		0	0	0
Left X	2.17		5.50	0	0
Right Crus I	1.93		0	0	0
Right Crus II	3.38		0	2.78	0
Right I-III	1.69		0	5.56	5.00
Right IX	0.24		0.92	0	0
Right V	0.97		0.92	0	0
Right VI	1.93		0.92	0	**15.00**
Right VIIB	1.21		0.92	0	0
Right VIIIA	4.59		5.50	8.33	0
Right VIIIB	2.66		2.75	5.56	0
Right X	0.97		0	0	0
Right IV	3.14		3.67	0	0
Vermis IX	2.17		0.92	0	0
Vermis.VI	0.72		0.92	0	0
Vermis VII	1.93		1.83	2.78	0
Vermis VIII	4.59		1.83	5.56	5.00
Vermis	0.24		0	0	0
Total Cerebellar Volume		5.80	0	2.78	0

Deviations in > 10% of the individuals in bold

HC: healthy controls; SSD-NV: non-violent schizophrenia spectrum disorder; SSD-V: schizophrenia spectrum disorder with a history of severe violence; NPV: non-psychotic violent offenders

**Table 7:** Negative deviations (< − 2 Z) in percent

Lobule	HC	SSD-NV	SSD-V	NPV
Left Crus I	0.24	3.67	2.78	5.00
Left Crus II	1.21	2.75	2.78	0.00
Left I-III	2.17	2.75	2.78	0.00
Left IV	0.97	4.59	5.56	5.00
Left IX	0.72	5.50	5.56	0.00
Left V	1.45	3.67	5.56	10.00
Left VI	1.45	6.42	0.00	10.00
Left VIIB	1.69	3.67	8.33	10.00
Left VIIIA	1.93	5.50	5.56	10.00
Left VIIIB	0.72	6.42	2.78	**15.00**
Left X	2.42	2.75	2.78	0.00
Right Crus I	1.69	1.83	5.56	10.00
Right Crus II	1.69	7.34	5.56	10.00
Right I-III	3.86	3.67	0.00	5.00
Right IX	2.90	2.75	8.33	10.00
Right V	2.66	1.83	8.33	0.00
Right VI	1.21	2.75	5.56	5.00
Right VIIB	0.24	0.00	8.33	5.00
Right VIIIA	0.72	0.00	2.78	0.00
Right VIIIB	2.42	7.34	5.56	5.00
Right X	3.14	5.50	5.56	**20.00**
Right IV	1.45	1.83	2.78	5.00
Vermis IX	1.45	6.42	**11.11**	5.00
Vermis VI	3.62	3.67	5.56	**15.00**
Vermis VII	2.17	3.67	2.78	0.00
Vermis VIII	0.24	3.67	2.78	0.00
Vermis X	5.31	5.50	5.56	**15.00**
Total Cerebellar Volume	0.24	1.83	8.33	**15.00**

Deviations in > 10% of the individuals in bold

HC: healthy controls; SSD-NV: non-violent schizophrenia spectrum disorder; SSD-V: schizophrenia spectrum disorder with a history of severe violence; NPV: non-psychotic violent offenders

## Discussion

In this study, we investigated associations between schizophrenia spectrum disorders, violent behaviour, and cerebellar volumes using novel cerebellar subregion segmentation methods and normative modelling of individual volume deviation patterns. The main findings were 1) decreased grey matter volumes in the posterior cerebellar hemispheres and the posterior vermal regions in schizophrenia independent of a history of severe violence, but with a different subregional distribution, and 2) a more widespread pattern of negative deviations from the norm at the individual level associated with severe violence.

As expected, persons with schizophrenia independent of a history of severe violence showed significantly lower volume in the total cerebellum compared to healthy controls. In line with our hypothesis, volume reductions on a subregional level in both patient groups appeared in the posterior cerebellum, specifically in the lobules left Crus I and right Crus II (persons with schizophrenia with no history of violence (SSD-NV)), and right Crus I (SSD-V), respectively. This is in line with earlier studies where decreased left Crus I volumes in schizophrenia have been shown in cerebellum optimised high resolution structural MRI studies [51–53], and a meta-analysis of drug-naïve first episode schizophrenia patients where volume decrease specifically in the left Crus I was found [54]. Moreover, decreased volume in the posterior vermis is in accordance with previous studies in men with schizophrenia [55]. Furthermore, grey matter volume (GMV) alterations in the cerebellar vermis, predominantly in posterior subregions, in patients with schizophrenia were confirmed in a recently published systematic review [56]. The consistency with earlier findings of cerebellar subregional abnormalities in schizophrenia supports the representativity of the current sample and methods and points towards a distinct pattern of cerebellar subregion morphometry in schizophrenia across illness stages.

When it comes to violence and conditions related to violence, e.g., impulsivity, anger, and psychopathy, the most consistent findings from earlier studies are GMV decreases in the right posterior cerebellum (which Crus I is a part of) and the vermis [8]. It should, however, be stressed that our study did not reveal results which would allow to separate the SSD groups by a history of violence.

In the present study, a substantial part of SSD-V showed individual-level extreme negative deviations in the Vermis IX region, which is consistent with the results from the group comparison analyses. There were, however, no clearly coinciding results between the group analyses and the normative modelling regarding the other subregions. This reflects that the two methods provide complemental information. Interestingly, several violent offenders without psychosis (NPV) showed extreme volume deviations, mostly negative (in subregions left lobule VIIIB, right lobule X, Vermis VI and Vermis X), but also positive in the subregion lobule VI bilaterally. These findings may represent a greater heterogeneity within the NPV group and reflect the more widespread findings of both volume decreases and, to a lesser degree, increases in the context of violence, aggression and impulsiveness [8].

In the group comparisons, we found no statistically significant differences in cerebellar volume between persons with a history of violence without schizophrenia and healthy controls. This is somewhat in contrast to previous studies showing either negative [8] or positive correlations between cerebellar volumes in violent offenders [57] and psychopathic traits in non-psychotic violent offenders [58] compared to non-violent healthy controls. However, by applying normative models, we found several deviations from the norm within this group. The distribution of extreme volume deviations in the NPV group showed a heterogenous pattern, which may reflect the complexity of the violence construct, diversity among the NPV cohort, or multifaceted biological underpinnings. Further, this complexity underscores the importance of studying brain morphological characteristics at the individual level.

Regarding possible functional correlates of the reported volumetric changes,

smaller whole and regional cerebellar volumes have been associated with low premorbid cognitive functioning in psychosis patients [59]. Volume reductions specifically in the vermal region IX and right crus I have been reported in a meta-analysis carried out in 363 individuals (323 males) with Autism spectrum diagnosis (ASD) [60]. The vermis projects predominantly to limbic, reticular and autonomic regions [61]. The vermal region of lobule IX plays a role in emotion processing and assignment of facial expression [62]. A decrease in volume in this part of the vermis may point to the impairment of the automatic or implicit processing of mentalizing, which is crucial for understanding the actions and intentions of others [63]. Hence, it is conceivable that this part of the vermis may represent an important part of the morphological substrate of cognitive dysmetria [12]. Moreover, vermis and right posterior cerebellum have been related to increased impulsivity [21]. Transcutaneous vagal stimulation has shown to increase blood flow in the bilateral Crus I and the vermal IX regions, eliciting their function for mediating autonomous responses [15]. Interestingly, stimulating the rostral vermis by an implanted pacemaker reduced aggressive and violent behaviour in patients with chronic severe schizophrenia spectrum disorders [64]. The lobules Crus I and Crus II are correlated with the default mode network (DMN) and may hence be involved in emotional and social processes, self-referential thought, and mentalizing [15]. A correlation between severity of ASD core symptoms in social interaction and communication and reduced GMV in right Crus I and II has been reported [65]. Of note, the ability to identify and relate to mental states of others, (i.e., theory of mind), is impaired in both ASD and SSD [66, 67]. Moreover, the cerebellum presumably has an important role in understanding sequences and making predictions [68, 69] which may play a mediating role in violent behaviour in some SSD patients insofar as incorrect or strongly biased predictions might cause frustration, perception of being threatened, and anger, resulting in physical violence. Finally, reduced volume of the cerebellar vermis and left hemisphere is not confined to individuals with SSD but has also been shown in patients with PTSD [70]. Interestingly, the relationship between traumatic experiences and violence in SSD patients is well established [71]. Taken together, the present results may indicate an association between (premorbid) traits of neurodevelopmental disorder in subregions of the cerebellum, which seem to play an important role in mentalizing and the implicit coordination of social behaviour and increased proneness to violence in some SSD patients. As such, our findings also may comply with a unitary model of schizophrenia [13].

One limitation of the current study is the relatively low number of individuals in the SSD-V and the NPV group, which reflects the difficulty of recruiting these groups due to severe psychopathology, security considerations, and the ability to give informed consent. It should, however, be taken into account that the entire population of men serving a preventive detention sentence in Norway was only 120 [72] during study inclusion. The unexpected absence of significant volume differences between the SSD-V, SSD-NV, and NPV group could reflect a true null finding. However, we cannot exclude the possibility of a Type II error. Achieving satisfactory power is described as a common challenge in the field of neuroimaging studies [73], and an inherent limitation in studies comprising rare phenotypes. To overcome the limitations of small subject samples, we applied normative modelling which has been developed for this purpose, that is to explore brain volumetric characteristics at the individual level and in comparisons with a large reference population. Furthermore, MRI data were obtained by two different scanners. While this could potentially introduce variability, the adaptation and usage of normative models have been shown to effectively account for this issue [74]. Moreover, the normative models target deviations from the norm at the individual level, which is a valuable approach in small subject samples. An additional limitation of our study is that we did not differentiate between grey and white matter composition within the cerebellar regions, which may limit direct comparability with studies focusing on these specific tissue types. However, since we used openly accessible cerebellar normative models [32], future studies employing the same approach will be able to directly compare their findings with ours, thereby enhancing consistency and comparability across research in this field.

Notwithstanding this, the study has several strengths: First, the thorough clinical assessments, including highly reliable characterization of patients with a history of severe violence and absence of violence, respectively. Secondly, the high application of state-of the art neuroimaging methodology including normative modelling and cerebellar subregion segmentation. Finally, the use of two complemental methods may be a more appropriate approach to take into account the complexity of volume alterations when exploring violence in psychosis than comparing group means only.

## Conclusion

We report smaller grey matter volumes in subregions of the posterior cerebellar hemispheres and the posterior vermis in men with schizophrenia spectrum disorders, but with a different subregional distribution in patients with and without a history of violence. Although not significantly separating persons with schizophrenia with or without a history of violence, and in need of replication, the results are in line with earlier studies suggesting that disrupted fine-tuning of social behaviour is of relevance for violence in psychosis. Moreover, the partially converging results from the group comparisons and the normative modelling analyses demonstrate the usefulness of complemental methodological approaches to disentangle the complex biological associations between violence and psychosis.

Our results may contribute to increased understanding of the underpinnings of violent behaviour in some patients with schizophrenia spectrum disorders.

## Data Availability

This study used the Services for Sensitive Data (TSD), University of Oslo, Norway, with resources provided by UNINETT Sigma2—the National Infrastructure for High-Performance Computing and Data Storage in Norway. Due to ethical and data security issues related to the sensitive nature of the clinical data, we are not allowed to share the data without specific IRB approval and data use agreements with the relevant institution.
